# Distribution of PACAP and PAC1 Receptor in the Human Eye

**DOI:** 10.1007/s12031-022-01985-0

**Published:** 2022-03-07

**Authors:** Evelin Patko, Edina Szabo, Denes Toth, Tamas Tornoczky, Inez Bosnyak, Alexandra Vaczy, Tamas Atlasz, Dora Reglodi

**Affiliations:** 1 Department of Anatomy, Medical School, MTA-PTE PACAP Research Team, University of Pecs, 7624 Pecs, Hungary; 2 Department of Forensic Medicine, Medical School, University of Pecs, 7624 Pecs, Hungary; 3 Department of Pathology, Medical School and Clinical Center, University of Pecs, 7624 Pecs, Hungary; 4 Szentagothai Research Center, Medical School, University of Pecs, 7624 Pecs, Hungary; 5 Department of Sportbiology, University of Pecs, 7624 Pecs, Hungary

**Keywords:** Human eye, PACAP, PAC1 receptor, Immunohistochemistry

## Abstract

Pituitary adenylate cyclase–activating polypeptide (PACAP) is a neuropeptide with widespread distribution and diverse biological functions. Several studies show that PACAP has strong cytoprotective effects mediated mostly through its specific PAC1 receptor (PAC1-R) and it plays important roles in several pathological conditions. Its distribution and altered expression are known in various human tissues, but there is no descriptive data about PACAP and its receptors in the human eyebulb. Since PACAP38 is the dominant form of the naturally occurring PACAP, our aim was to investigate the distribution of PACAP38-like immunoreactivity in the human eye and to describe the presence of PAC1-R. Semiquantitative evaluation was performed after routine histology and immunohistochemical labeling on human eye sections. Our results showed high level of immunopositivity in the corneal epithelium and endothelium. Within the vascular layer, the iris and the ciliary body had strong immunopositivity for both PACAP and PAC1-R. Several layers of the retina showed immunoreactivity for PACAP and PAC1-R, but the ganglion cell layer had a special pattern in the immunolabeling. Labeling was observed in the neuropil within the optic nerve in both cases and glial cells displayed immunoreactivity for PAC1-R. In summary, our study indicates the widespread occurrence of PACAP and its specific receptor in the human eye, implying that the results from in vitro and animal studies have translational value and most probably are also present in the human eye.

## Introduction

Pituitary adenylate cyclase–activating polypeptide (PACAP) is a neuropeptide with widespread occurrence in the body. It has two forms, PACAP27 and PACAP38, consisting of 27 and 38 amino acids, respectively. In mammalian tissues, PACAP38 is the dominant form. PACAP binds to its specific PAC1 receptor (PAC1-R), and to VPAC1 and VPAC2 receptors, which also bind the closest homolog peptide, vasoactive intestinal peptide (VIP) with equal affinity (Moody et al. 2021; Vaudry et al. 2009). PACAP has very diverse actions depending, among others, on the receptor expression pattern of the different tissues. PACAP plays a role in neuronal excitability (May et al. 2021), urinary bladder activity (Girard et al. 2021), gastrointestinal motility and secretion (Karpiesiuk and Palus 2021; Reglodi et al. 2018; Rytel et al. 2021), cartilage and bone formation (Jozsa et al. 2021), and reproduction and embryonal growth (Koves et al. 2020; Ross et al. 2018; Shan et al. 2021) and it also has immunomodulatory functions (Abad and Tan 2018). Several studies show that PACAP plays important roles in numerous pathological conditions such as tumor growth and proliferation (D’Amico et al. 2021b, c; Maugeri et al. 2018a, 2021; Moody et al. 2016) and nervous system disorders (Moody and Jensen 2021) like migraine, schizophrenia, anxiety, and depression (Eslami et al. 2021; Kormos et al. 2016; Martelle et al. 2021; Ross et al. 2020; Tiihonen et al. 2021) as well as inflammatory conditions (Moody and Jensen 2021; Tamas et al. 2021), sudden infant death syndrome (Shi et al. 2021), and hearing loss (Fulop et al. 2019; Ruel et al. 2021).

One of the most intensively studied actions of PACAP is its neuroprotective and general cytoprotective effect. This has been demonstrated in numerous in vitro and in vivo studies (Reglodi et al. 2011, 2017). Recently, it has been shown that in addition to the long-known protective effects in models of stroke and Parkinson’s disease (Ohtaki et al. 2008; Zheng et al. 2021), PACAP is protective in models of spinal and bulbar muscular atrophy (Martinez-Rojas et al. 2021), fetal alcohol syndrome (Shili et al. 2021), diabetic neuropathy (Kiss et al. 2021), optic neuritis in multiple sclerosis (Van et al. 2021), and noise-induced hearing loss (Ruel et al. 2021). The large body of evidence, showing that PACAP is protective in animal models of several diseases, places PACAP on the list of emerging protective therapeutic agents in neurodegenerative disorders (Cheng et al. 2020; Soles-Tarres et al. 2020) and stroke (Cherait et al. 2021; Fang et al. 2020; Sadanandan et al. 2021; Zheng et al. 2021).

PACAP is also present in the eye, in several retinal layers as well as iris, cornea, and ciliary body displaying immunoreactivity for both the peptide and its receptors (Wang et al. 1995). Numerous actions have been described in ocular tissues (D’Amico et al. 2021a; Postyeni et al. 2021). Among others, PACAP stimulates tear production and prevents corneal hyperkeratinization (Nakamachi et al. 2016); it is necessary for stable pupil maintenance (Keenan et al. 2016) and it plays an important role in the regulation of circadian rhythm via the retinohypothalamic pathway (Vereczki et al. 2006). The general protective effects of PACAP can also be observed in the eye. Although some data indicate that PACAP may provoke inflammatory reactions in the rabbit eye (Wang et al. 1997), most available data indicate that PACAP has very potent protective effects in the retina and cornea. For example, PACAP is protective in diabetic, ischemic, inflammatory retinopathies, in retinopathy of prematurity, in glaucoma (Atlasz et al. 2010; Kvarik et al. 2021; Maugeri et al. 2019a; Szabo et al. 2021; Vaczy et al. 2018), as well as traumatic and excitotoxic retinal injuries (Atlasz et al. 2009; Seki et al. 2008).

PACAP and its receptors occur in ocular tissues in different species, such as rabbit, rat, and mouse (Troger et al. 2007; Wang et al. 1995). As the role of PACAP is emerging also in human diseases as a biomarker and an increasing number of data support the functions of PACAP in human tissues, it is essential to investigate the distribution of PACAP and its receptors in the human eye. However, there are very few data regarding the occurrence and actions of PACAP in the human eye. Olianas and coworkers (2002) have reported that PACAP increases cAMP levels in fetal retinas and could demonstrate the presence of the mRNA of PACAP and of its receptors in retinal homogenates. Pigment epithelial and corneal endothelial cells derived from human eyes have been subject of a series of in vitro investigations that show that PACAP stimulates adenylate cyclase, protects the cells against growth factor deprivation, oxidative stress, or hyperglycaemia, and stimulates various intracellular signaling pathways (Fabian et al. 2012, 2019; Maugeri et al. 2017, 2018b, 2019a, b). Retinoblastomas have also been shown to express PACAP receptors (Olianas et al. 1996), where, interestingly, PACAP acts as a cytotoxic agent in high concentrations (Wojcieszak and Zawilska 2014). However, there are no further data available in the normal human eye. Therefore, the aim of the present study was to describe the distribution of PACAP-like immunoreactivity in the human eye. As the protective effects of PACAP are predominantly mediated by its specific PAC1-R, we also studied the presence of PAC1 receptor in different parts of the human eye.

## Materials and Methods

### Human Samples

Human eyes (*N* = 7 patients; 6 boys, 1 girl) were used in this experiment (ethical permission No: 6383-PTE 2018). The age of patients, undergoing enucleation surgery because of retinoblastoma, was 16 ± 10 months. Only the tumor-free, normal parts were used for histological analysis. Tissues were fixed in 10% neutral buffered formalin, dehydrated in graded alcohol series, embedded in paraffin, cut in 3-µm-thick sections with a rotational microtome (Microm HM 325, Thermo Scientific, Ltd.), and mounted on coated glass microscope slides. After deparaffinization and rehydration, samples were pretreated with heat-induced epitope retrieval method in 1 mM (pH = 6.0) citrate buffer in a microwave oven for 15 min at 750 W. After cooling on room temperature, tissues were washed in TRIS buffered saline solution (TBS) (pH = 7.6).

### Immunohistochemistry Analysis

For immunohistochemistry, samples were incubated in anti-PAC1-R antibody (Cat. Nr. AVR-003, Alomone Labs, Ltd., 1:125, 1 h at room temperature), and anti-PACAP38 (Cat. Nr. T-4473, BMA Biomedicals, Ltd., 1:500, 1 h at room temperature). Sections were washed in TBS and incubated with HISTOLS-AP-R anti-rabbit alkaline phosphatase labelled detection system (Cat. Nr. 30,011.R500A, Histopathology, Ltd., 30 min at room temperature). After washing in TBS, the reaction was developed with HISTOLS Resistant AP-Red Chromogen/substrate System (Cat. Nr. 30,019, Histopathology, Ltd.) in a dark environment. Staining intensity was controlled under light microscope after 10 min of incubation with the chromogen/substrate working solution. Our choice was this chromogen substance for its magenta staining, so positive immunoreaction would also be visible in the pigmented cells. Sections were counterstained with hematoxylin solution, and bluing was performed with tap water. Samples were dehydrated in alcohol, cleared in xylene, and mounted with permanent mounting medium. Negative control was obtained when the primary antibody was replaced with TBS. The slides were digitalized using a Panoramic MIDI II automatic digital slide scanner (3DHISTECH Ltd., Hungary) and images were taken with CaseViewer 2.3 software (3DHISTECH Ltd., Hungary). Sections were analyzed using a semiquantitative approach. Immunoreactivity was scored by 3 researchers, between 0- +-+ +-+ + + depending on the staining intensity.

## Results

**Fig. 1:**
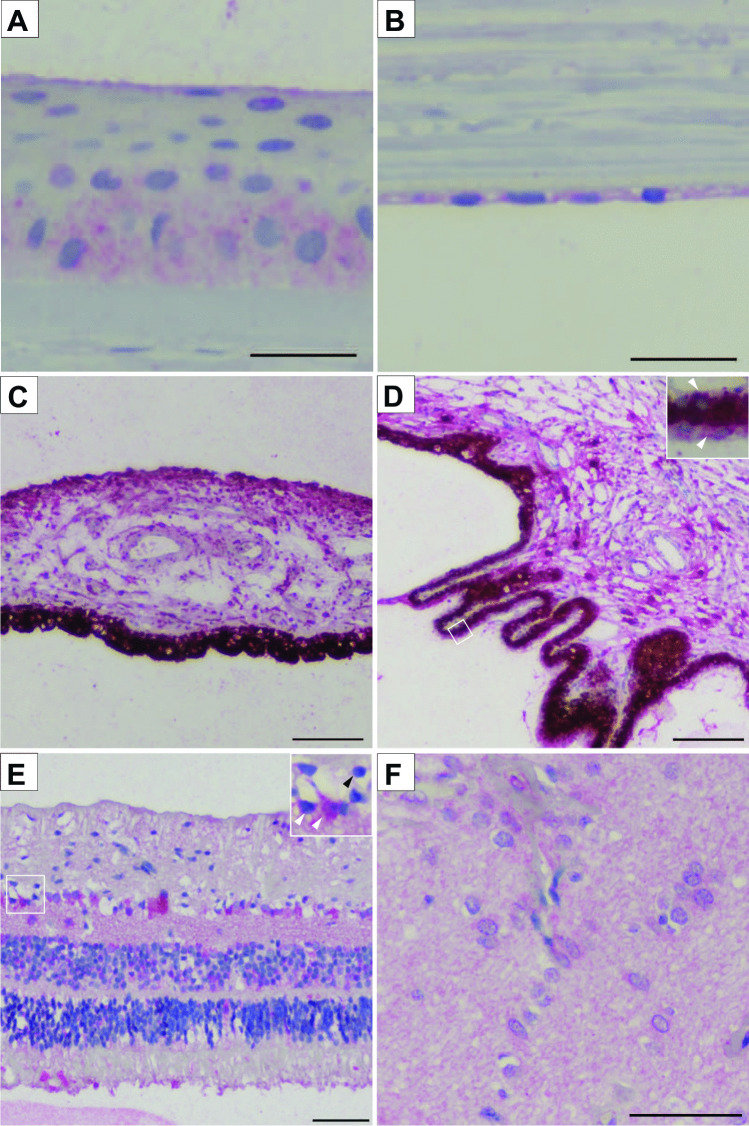
Representative light microscopic pictures of the magenta-stained PAC1-R positive areas in the human eyes. **A** Corneal epithelial cells showed PAC1-R immunopositivity. **B** Corneal endothelial cells were strongly PAC1-R positive. **C** In the iris, the pigmented epithelial cells and stroma showed strong immunopositivity. **D** The ciliary body displayed strong positivity at the bases of the ciliary processes and weak immunosignal was found in the ciliary muscle. Pigmented epithelial cells showed strong PAC1-R positivity with high magnification (white arrowheads in inset) **E** Several retinal layers displayed high PAC1-R immunosignal. In high magnification, PAC1-R positive (white arrowheads) and PAC1-R negative (black arrowhead) cells are shown in the ganglion cell layer. **F** The optic nerve showed moderate immunoreactivity for PAC1-R in the neuropil, while glial cells had strong immunopositivity. Scale bar: 50 µm (**A**, **B**, **E**, **F**); 100 µm (**C**, **D**)

**Fig. 2:**
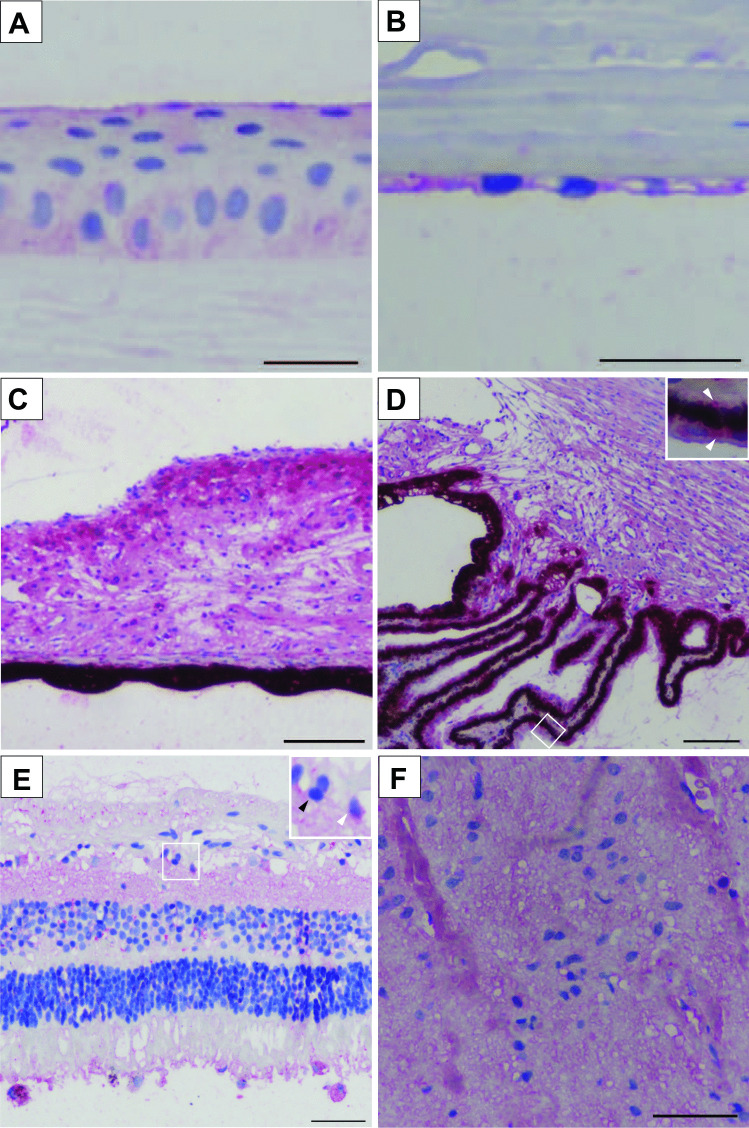
Representative light microscopic pictures of the magenta-stained PACAP positive areas in the human eyes. **A** Epithelial basal layers of the cornea showed strong immunosignal. **B** Endothelial layer of the cornea was strongly positive for PACAP. **C** In the iris, the stroma, sphincter pupillary muscle showed strong immunoreaction; the dilator pupillary muscle had positivity only in the posterior part. **D** The ciliary stroma showed weaker staining. Inset highlights the pigmented epithelium where high immunosignal was shown (white arrowheads in inset). **E** PACAP positivity was observed in the retinal nerve fiber layer, ganglion cell layer, inner plexiform, and nuclear layers and also in the pigmented epithelium. Inset highlights the PACAP positive ganglion cells (white arrowhead) and PACAP negative ganglion cells (black arrowhead) distribution within the ganglion cell layer. **F** Optic nerve showed moderate immunoreactivity in the neuropil but glial cells did not show PACAP immunosignal. Scale bar: 50 µm (**A**, **B**, **E**, **F**); 100 µm (**C**, **D**)

Results are summarized in Table 1. The outer layer of the eyebulb, the tunica fibrosa or fibrous layer, consists of the sclera and cornea. The sclera is a dense connective tissue layer, which was negative for both PACAP and its PAC1-R. The cornea, on the other hand, displayed positive areas. The cornea has an outer epithelial layer made up of stratified squamous non-keratinized epithelium and an inner endothelial layer, made up of a single layer of simple squamous cells, the endothelial cells, facing the anterior chamber. Between the epithelial layers, the corneal stroma is found, separated by the limiting membranes from the outer and inner epithelium. While the stroma, which is similar in its main histological characteristics to those of the sclera, remained negative, the epithelial cells showed immunopositivity for both PACAP and PAC1-R. The outer epithelium showed strong immunopositivity in the basal layers in all sections and in the upper, planocellular layer in some sections. The middle polygonal layer did not show positivity in any of the sections. These findings are indicated as 0/ + + in Table 1. The inner endothelial layer was strongly positive in all cases (Figs. 1A, B; 2A, B).

**Table 1:** Localization and relative abundance of PACAP and PAC1-R immunoreactivity in the human eye. The symbols provide a semiquantitative evaluation of the density of PACAP and PAC1-R; + + + : high density; + + : moderate density; + : low density; 0: no signal. In the table “/” indicates the difference within one section in different parts of one layer, “-” indicates the different distribution of the immunopositivity between different sections

Structures			PACAP	PAC1-R
Fibrous layer	Cornea	Epithelium	0/ + +	0/ + +
		Stroma	0	0
		Endothelium	+ +	+ +
	Sclera		0	0
Vascular layer	Iris	Pigment epithelium	+ + +	+ + / + + +
		Stroma	+ + / + + +	+ + / + + +
		Sphincter pupillary muscle	+ / + +	0- +
		Dilator pupillary muscle	+ + +	0/ + +
	Ciliary body	Pigmented epithelium	+ + +	+ + +
		Non-pigmented epithelium	+ + +	+ +
		Stroma	+	+ / + + +
	Choroid		0	0
Nervous layer		Nerve fiber layer	+	+
		Ganglion cell layer	0/ + +	0/ + + +
		Inner plexiform layer	+ +	+ / + +
		Inner nuclear layer	0/ +	+
		Outer plexiform layer	0	0- +
		Outer nuclear layer	0	0- +
		Layer of rods and cones	0- +	0- +
		Retinal pigmented epithelium	+ +	+ + +
Optic nerve	Glia		0	+ +
	Neuropil		+ +	+ +

The middle layer of the eyebulb is the vascular layer, or uvea, which is composed of the iris, ciliary body, and choroid parts. The main tissue of the iris is the stroma, where the sphincter and dilator pupillary muscles are embedded. Behind the stroma, the blind part of the retina is located as a double layer of pigmented epithelial cells. The magenta-stained positive immunoreaction was visible for both PACAP and its receptor (Figs. 1C, 2C). The stroma showed strong immunoreactivity with both antibodies, but the distribution of the immunoreactivity was uneven: the anterior part of the stroma displayed stronger immunopositivity (indicated as + + / + + + in Table 1). Interestingly, the spinchter pupillary muscle was more positive for PACAP than for PAC1-R, which showed very weak positivity only in some sections (PAC1-R indicated as 0- + in Table 1). The dilator pupillary muscle was positive for PACAP in its entire length, while positive for PAC1-R only in the posterior part (indicated as 0/ + + in Table 1). The ciliary body stroma has the ciliary muscle embedded, and the anterior part of the ciliary shows projections called ciliary processes, which produce the aqueous humor. The posterior part of the ciliary is also covered by the continuation of the non-visual part of the retina as an outer pigmented and an inner non-pigmented layer (ciliary part of the retina). The non-pigmented retinal layer showed strong positivity for both PACAP and PAC1-R. The stroma was weakly stained for PACAP, while PAC1-R immunosignal was very strong at the bases of the ciliary processes and very weak in the ciliary muscle (Figs. 1D, 2D). The choroid was negative for the antibodies.

**Fig. 3:**
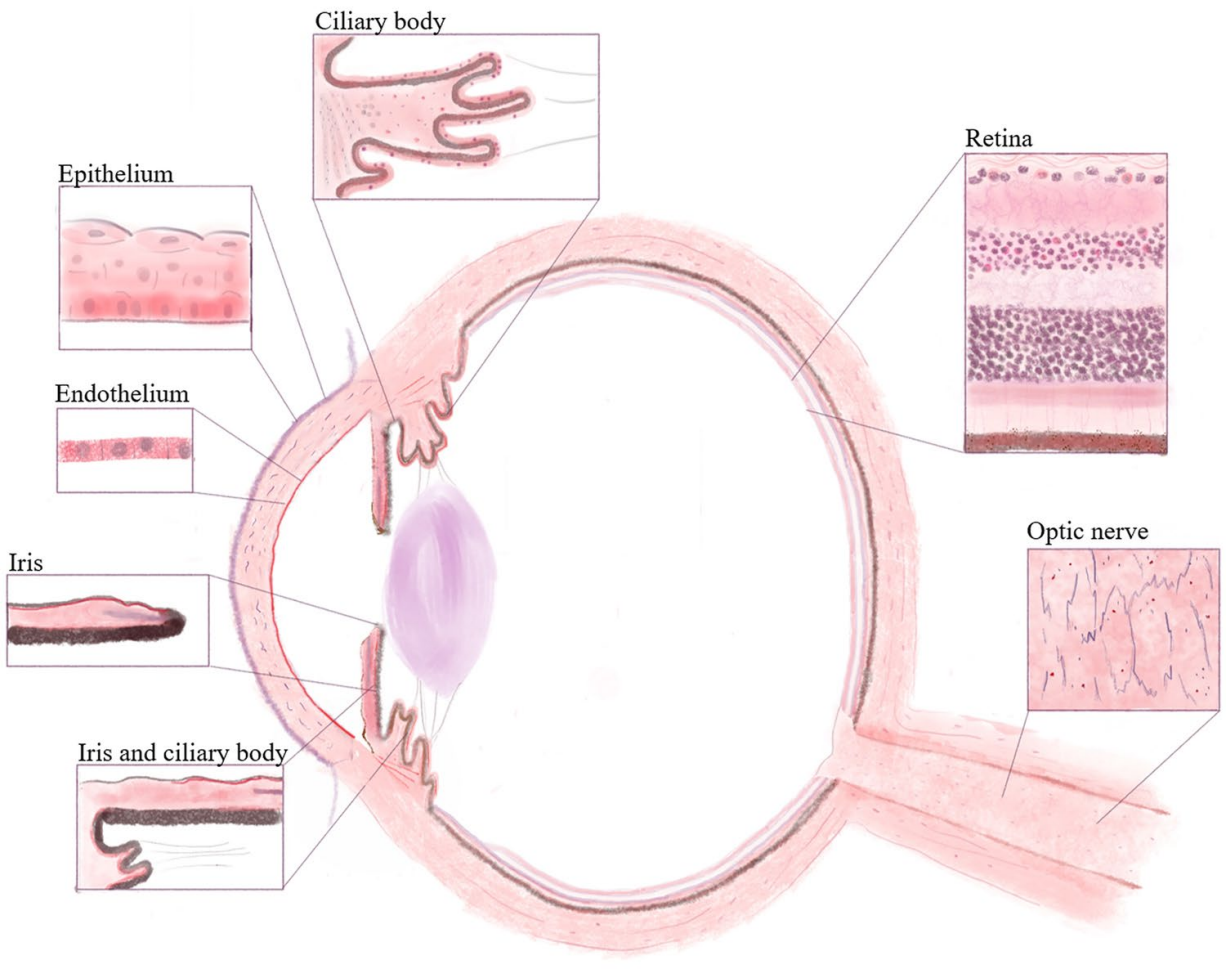
Schematic drawing of PACAP and PAC1-R distribution in the human eye. Red color indicates PACAP and PAC1-R-expressing cells according to our results. The highlighted areas represent the most relevant parts

The retina has 10 layers, the first (pigmented) layer developing from the original outer layer of the eyecup, while the other 9 layers derive from the original inner retinoblasts. Positivity for PACAP and very strong expression for PAC1-R were detected in the pigmented epithelial layer, where the magenta color was easily distinguishable from the brown pigmentation of the epithelial cells. The layer of the photoreceptors (rods and cones) showed weak or no immunostaining in an individually variable pattern (indicated as 0- + in Table 1), similarly to the outer nuclear and plexiform layers, which displayed a weak signal for PAC1-R only in some samples. The inner nuclear layer contains the cell bodies of the bipolar neurons, those of the retinal interneurons (amacrine and horizontal cells) and those of the retinal Müller glial cells. This layer was positive in most cases for both the peptide and the receptor. An interesting staining pattern was observed in the ganglion cell layer, where very strong immunosignal could be seen in some of the ganglion cells, while others were negative (indicated as 0/ + + + in Table 1, Figs. 1E, 2E). The optic nerve is formed from the axons of the retinal ganglion cells. As a projection of the diencephalon during development, it is not a peripheral nerve, but part of the central nervous system. Therefore, the optic nerve is covered by the meninges and contains glial cells of the central nervous system. In the optic nerve, we found moderate immunoreactivity for PACAP and PAC1-R in the neuropil, while the glial cells only displayed immunoreactivity for the receptor (Figs. 1F, 2F). Schematic representations of our findings are shown in Fig. 3.

## Discussion

In the present study, we described the distribution of PACAP and PAC1-R-like immunoreactivity in the human eye. We found immunopositivity in the corneal epithelium and endothelium, in the stroma and muscles of the iris and ciliary body. The retina displayed strong immunoreactivity in several layers, including the pigment epithelial cells, while the optic nerve had weaker immunoreactivity.

PACAP has been shown to occur in retinas and other ocular tissues of various species. Most studies have used rat and mouse retinas, where strong PACAP and receptor expression has been described (D’Agata and Cavallaro 1998; Denes et al. 2019). In mice, detailed mapping of PACAP and PACAP receptors is available by Seki and coworkers (Seki et al. 1997, 1998, 2000; Shioda et al. 2016). Similarly, PACAP and PAC1-R expression has been found in the chick retina, where PACAP expression shows circadian rhythm (Borba et al. 2005; Jozsa et al. 2001). PACAP has also been described in the turtle and fish retina (Grone et al. 2007; Reglodi et al. 2001). In several species, including monkeys, PACAP and melanopsin have been found to be co-stored in the melanopsin-containing retinal ganglion cells (Hannibal et al. 2014). Other parts of the eye have been less investigated. PACAP immunoreactivity has been described in the cat choroid (Elsås et al. 1996), while radioimmunoassay studies have revealed PACAP immunoreactivity in homogenates of the iris, ciliary body, cornea, retina, and choroid of the rabbit eye (Nilsson et al. 1994; Wang et al. 1995). Our study described, for the first time, the detailed distribution of PACAP and PAC1-R-like immunoreactivity in the human eye. The samples were from children under 3 undergoing enucleation surgery because of retinoblastoma. Therefore, the distribution could be different in adult eyes, but the eyebulbs used in our study showed fully developed ocular structures.

In the human retina, only Olianas and coworkers (2002) have reported that PACAP increases cAMP levels in fetal retinas and could demonstrate the presence of mRNA of PACAP and its receptors in retinal homogenates. We found that several layers, including the ganglion cell layer, express PACAP and its PAC1-R with a distribution pattern described in other mammalian species. Previously, it has been found that almost all intrinsically photosensitive melanopsin-containing ganglion cells express PACAP in mice, rats, and even in monkeys (Hannibal et al. 2014). These cells are thought to play a role in the transmission of light information for the centers responsible for generating circadian rhythm (Hannibal and Fahrenkrug 2004).

The retinoprotective effects of PACAP are widely known and have been proven by dozens of animal models and in vitro studies (Atlasz et al. 2016; Shioda et al. 2016). PACAP has also been shown to have a role against retinal aging, as early aging signs have been demonstrated in PACAP knockout animals (Kovacs-Valasek et al. 2017). Earlier it has been demonstrated that the melanopsin-containing retinal ganglion cells (which also express PACAP) are more resistant to degenerative processes, and this has raised the possibility of PACAP being involved in the endogenous protective machinery (La Morgia et al. 2011). Indeed, we have shown that mice lacking PACAP have increased vulnerability in models of retinal ischemia and retinopathy of prematurity (Kvarik et al. 2021; Szabadfi et al. 2012). The retinal pigment epithelial cells, as the first layer of the neural retina, play an important role in the photoprotection, metabolism, membrane renewal, vitamin A storage, and growth factor supply of the photoreceptors. Their involvement in several retinal diseases has been implied, such as diabetic retinopathy and age-related degeneration, and in vitro studies from human retinal pigment epithelial cells have described protection by PACAP against several harmful effects (Fabian et al. 2019; Maugeri et al. 2017, 2019a).

The pigment layer continues also in the blind part of the retina, where it is reflected in the iris to form two pigmented layers in the posterior border of the iris. In the posterior part of the ciliary body, the continuation of the retinal pigment epithelial cells forms the outer pigmented layer of the ciliary part of the retina, while a non-pigmented inner layer is derived from the embryonic inner layer of the retina. This latter structure builds the barrier between capillaries and the aqueous humor, thus playing an important role in the production of the aqueous humor. We found that both layers of the blind part of the retina express strong immunoreactivity for PACAP and its receptor. The presence of PACAP in the aqueous humor has been investigated in rabbit and human fluid samples, which showed that PACAP could not be detected under normal conditions, only after stimulation, when PACAP levels increased in the aqueous humor (Brubel et al. 2011; Wang et al. 1997). Although the direct involvement of PACAP in the aqueous humor production is not yet established, several lines of evidence support this hypothesis. cAMP is known to trigger transepithelial fluid transport across the ciliary epithelium in mammals (Cheng et al. 2016; Kong et al. 2006). As PACAP is a cAMP stimulating peptide, it can be assumed that the neuropeptide plays a role endogenously in the aqueous humor production. Furthermore, PACAP and its receptors have also been shown to act on chloride channels, which are essential in the production of aqueous humor, independently from the cAMP pathway (Alshafie et al. 2014; Derand et al. 2004; Leung et al. 2001; Martinez-Rojas et al. 2021). The role of PACAP has been implied not only in the production, but also in the absorption of the aqueous humor, as our most recent data have provided evidence that PACAP treatment leads to reduced intraocular pressure in a rat model of glaucoma (Szabo et al. 2021).

In addition to the pigmented epithelial cells of the iris and ciliary body, the stroma and the muscles also showed immunopositivity. Earlier studies have reported on the effects of PACAP on the intraocular smooth muscles. Yamaji et al. (2005) showed that PACAP enhanced sphincter response, but had no effect on the dilator pupillary muscle. The involvement of PACAP in the pupillary light reflex is also strengthened by the observation that both PACAP and PAC1 deficient mice have attenuated reflex (Engelund et al. 2012; Keenan et al. 2016). Although different, even contradictory data are available on the effect of PACAP on the iris muscles, a recent study has reported that the effect on the sphincter reflex depends on the light conditions, which might explain the reported differences (Keenan et al. 2016).

In addition to the inner and middle layers of the eye, we found strong immunoreactivity in the cornea part of the outermost, fibrous layer of the eye, where the outer epithelial and inner endothelial layers were positive for both the peptide and its receptor. PACAP treatment on the corneal surface has been shown to induce recovery of the epithelial cells and also of the sensory innervation (Fukiage et al. 2007; Ma et al. 2015; Wang et al. 2019). PACAP KO mice present dry eye symptoms with corneal hyperkeratinization, also pointing at the importance of endogenous PACAP (Nakamachi et al. 2016). Our finding that the endothelial cells display strong immunoreactivity for both PACAP and PAC1 receptors is in agreement with findings of Maugeri and coworkers (2019b), who showed the presence of PACAP and PAC1-R in corneal endothelial cells isolated from human corneal cells. PACAP’s protective effects have been confirmed in these cells (Maugeri et al. 2019b). Among others, PACAP showed protective effects against growth factor deprivation and induced epidermal growth factor receptor phosphorylation. These results show that PACAP may be an important factor in corneal integrity (Maugeri et al. 2018b, 2019b).

PACAP and/or its receptors have been shown in most human tissues, with the eye being an exception. In the present study, we provided evidence for the widespread occurrence of PACAP and its PAC1-R in the human eye. In human tissues, expression levels of PACAP and/or its receptors show alterations in various diseases. This has raised the question whether PACAP could be used as a biomarker for disease diagnosis and/or prognosis. Recent studies show diagnostic value of serum PACAP in non-traumatic osteonecrosis (Zhu et al. 2021), migraine (Yan et al. 2021), anxiety disorder (Ross et al. 2020), post-traumatic stress disorder (Wang et al. 2021), and multiple sclerosis (Al-Keilani et al. 2021). Tissue PACAP has also been suggested as a marker for tumor progression, like cervical cancer (Jung et al. 2011), tumors of kidney, testis, prostate and thyroid gland, pancreas, and large intestine (Bardosi et al. 2016; Ferencz et al. 2019; Godlewski and Łakomy 2010; Lindner et al. 2021; Nakamura et al. 2014; Szanto et al. 2012; Tamas et al. 2016). A recent human study investigating the transcriptomic profile of skin samples from patients undergoing carpal tunnel decompression surgery indicated that PACAP gene was the most strongly upregulated gene and its expression correlated with nerve fiber regeneration further suggesting a therapeutic potential in using PACAP for nerve regeneration (Baskozos et al. 2020; Maugeri et al. 2020a, b). All these studies draw attention to the importance of PACAP in human tissues. Very limited data had been available on the occurrence and almost no data on the distribution of PACAP and its receptors in the human eye. As dozens of studies have described different effects of PACAP in the eye, our study indicating the widespread occurrence of PACAP and its specific receptor in the human eye implies that the in vitro cellular effects and in vivo results from animal studies have translational value and most probably are also present in the human eye.

## Data Availability

The data presented in this study are available on request from the corresponding author.
